# Supplementary figures and images for: Identification of MOS9 as an interaction partner for chalcone synthase in the nucleus
Source: PeerJ. 2018 Sep 19;6:e5598. doi: 10.7717/peerj.5598 (PMC6151112; doi:10.7717/peerj.5598)

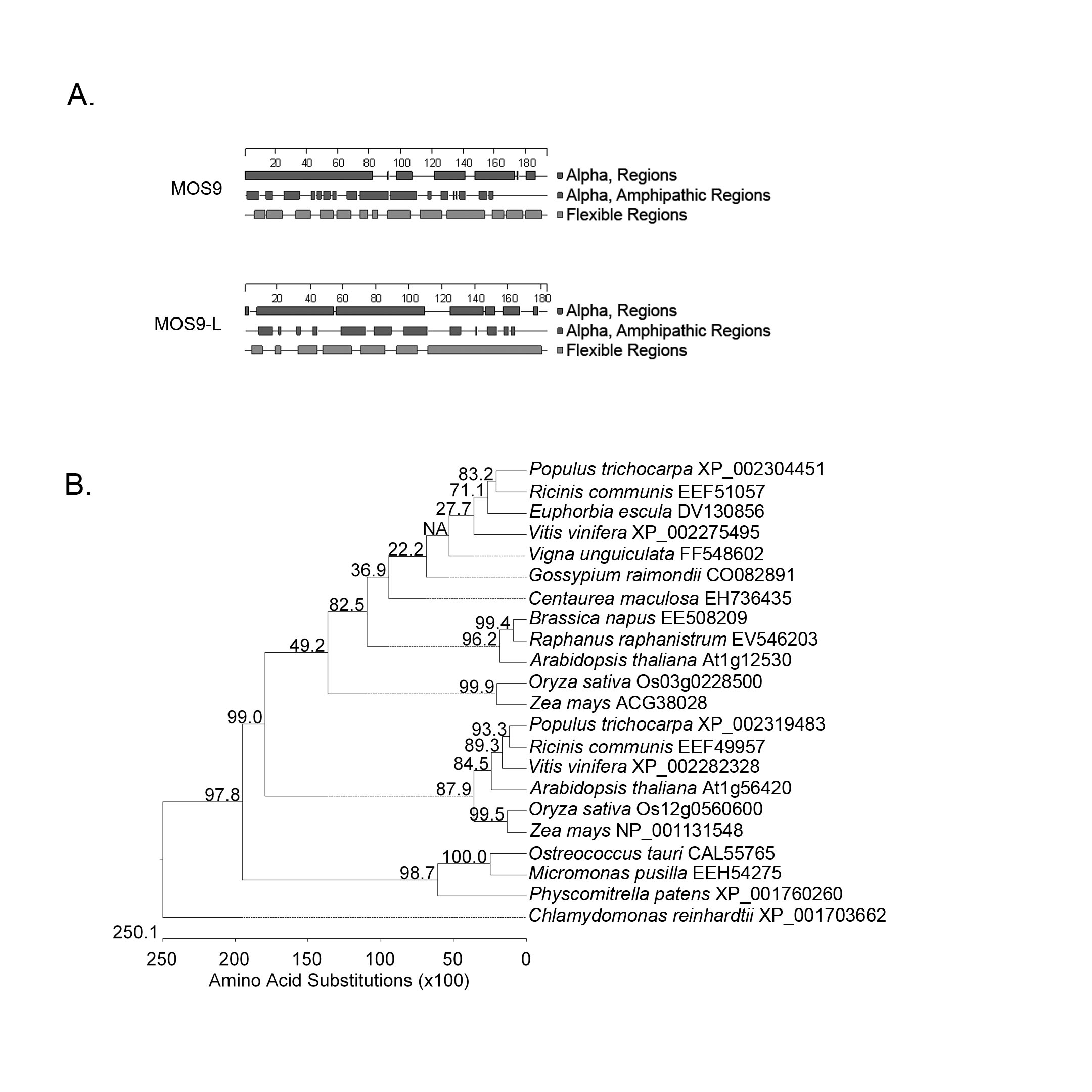

Supplement: Supplemental Information 1 — A) Secondary structure analysis performed using the Protean module in Lasergene (DNAStar, Madison, WI) showing predicted alpha helices and amphipathic alpha helical regions in the two proteins; similar results were obtained using I-Tasser (Yang and Zhang, 2015). B) Protein sequences with similarity to MOS9 were identified using tBLASTn on the NCBI Entrez interface. Phylogenetic analysis was accomplished using ClustalW of the MegAlign program of Lasergene with bootstrapping (1000 iterations). Phylogenetic relationships illustrate two distinct clades defined by homologs of the two proteins, plus a third clade comprising a truncated form present in moss and green algae. Yang JY, and Zhang Y. 2015. I-TASSER server: new development for protein structure and function predictions. Nucleic Acids Research 43:W174-W181. 10.1093/nar/gkv342 [file peerj-06-5598-s001.jpg]

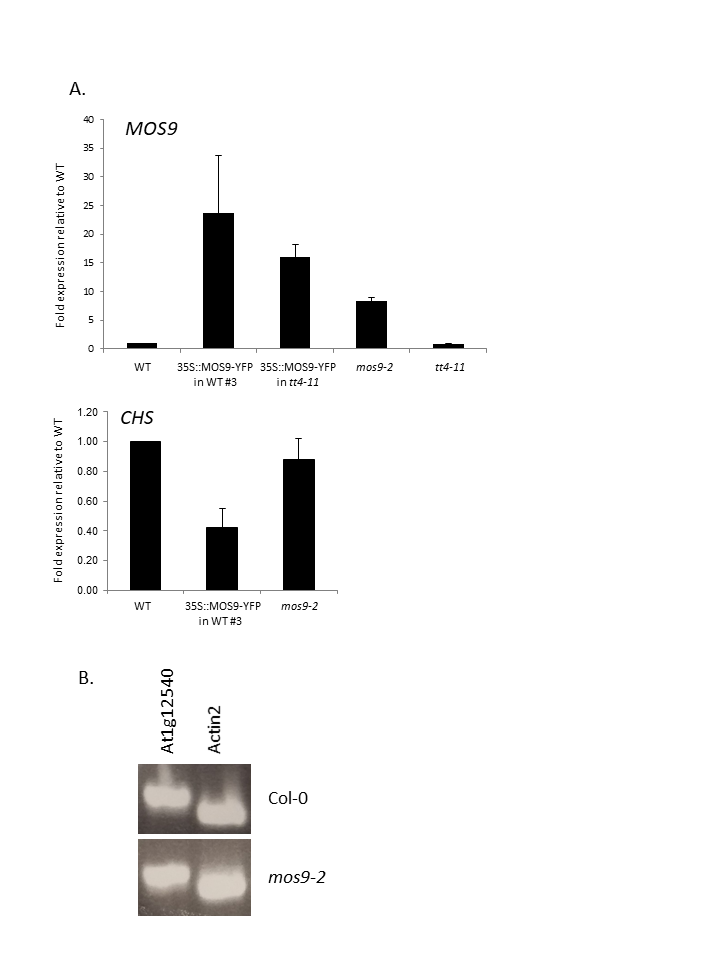

Supplement: Supplemental Information 3 — A) qRT-PCR analysis of MOS9 and CHS expression in various lines used in this study relative to the gene for ubiquitin-conjugating enzyme 21 (UBC). B) Semi-quantitative PCR analysis of expression of At1g12540 in wild-type and mos9-2 seedlings. [file peerj-06-5598-s003.png]
