# Supplementary material for: Identification of MOS9 as an interaction partner for chalcone synthase in the nucleus
Source: PeerJ. 2018 Sep 19;6:e5598. doi: 10.7717/peerj.5598 (PMC6151112; doi:10.7717/peerj.5598)
Supplement: Supplemental Information 2 — Comparisons are of CHS (At5g13930; red circles in B, C, and D), MOS9 (At1g12530; blue circles), RPP4 ( AT4G16860 ; green circles), and SNC1 ( AT4G16890 ; brown circles) in Arabidopsis tissues and organs (A) and in response to abiotic (B) and biotic (C and D) stress. Signal intensities are the average of data generated using the Affymetrix Arabidopsis ATH1 genome array analyzed using GENEVESTIGATOR® (Zimmerman et al. 2004). Data were filtered for 2-fold differences in expression values with p<0.001 for CHS (B), 1.5-fold and p<0.01 for CHS (C), and 1.5 fold and p<0.05 for MOS9; expression levels are presented as absolute signal values (A and B) or fold change ratios (C and D). Zimmermann P, Hirsch-Hoffmann M, Hennig L, and Gruissem W. 2004. GENEVESTIGATOR. Arabidopsis microarray database and analysis toolbox. Plant Physiology 136:2621-2632. doi.org/10.1104/pp.104.04636 [file peerj-06-5598-s002.pdf]

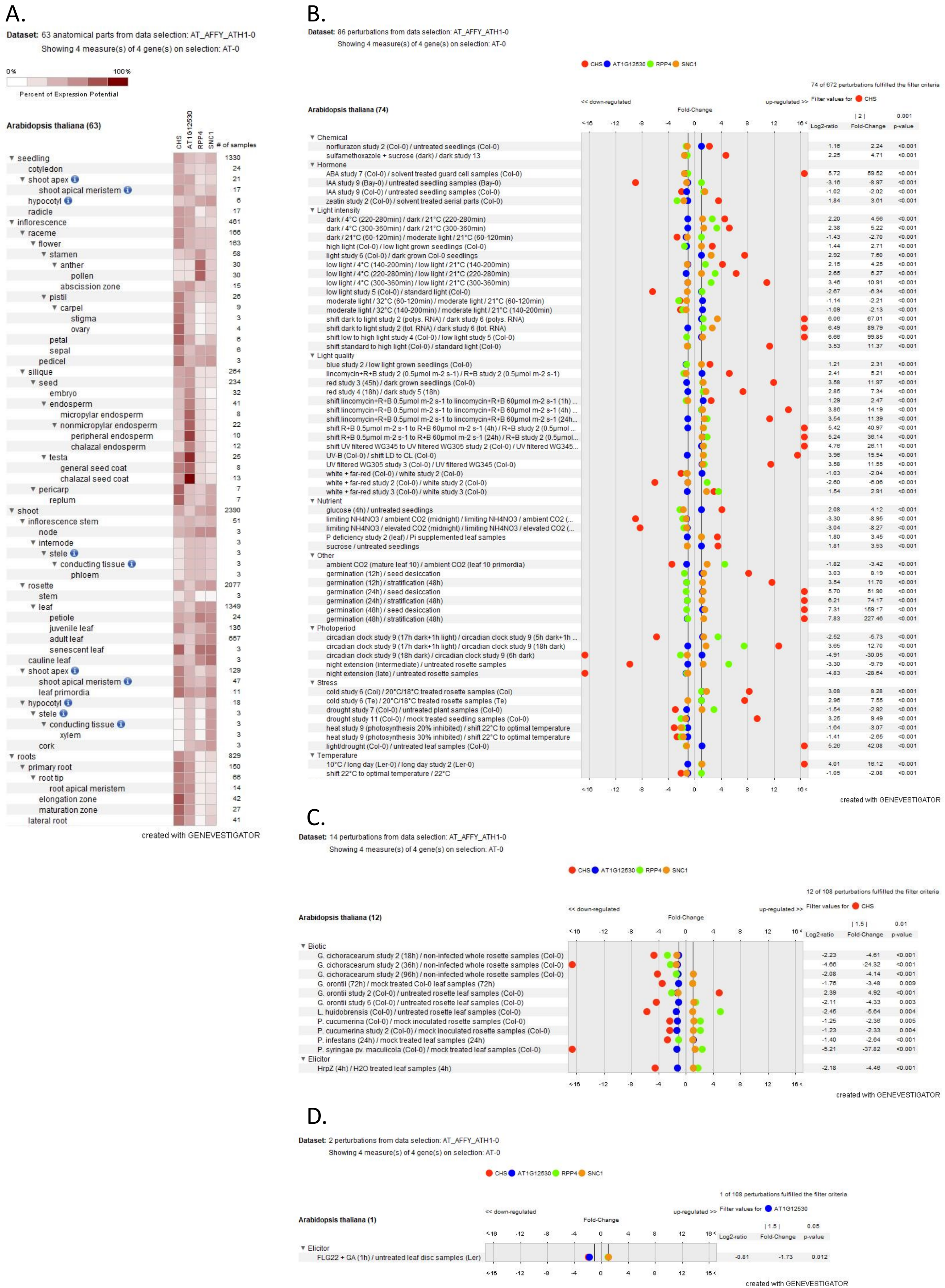
A.
Dataset: 63 anatomical parts from data selection: AT_AFFY_ATH1-0
Showing 4 measure(s) of 4 gene(s) on selection: AT-0
0%
100%
Percent of Expression Potential
Arabidopsis thaliana (63)
CHS
AT1G12530
RPP4
SNC1
# of samples
seedling 1330
cotyledon 24
shoot apex 21
shoot apical meristem 17
hypocotyl 6
radicle 17
inflorescence 461
raceme 166
flower 163
stamen 58
anther 30
pollen 30
abscission zone 15
pistil 26
carpel 9
stigma 3
ovary 4
petal 6
sepal 6
pedicel 3
silique 264
seed 234
embryo 32
endosperm 41
micropylar endosperm 8
nonmicropylar endosperm 22
peripheral endosperm 10
chalazal endosperm 12
testa 25
general seed coat 8
chalazal seed coat 13
pericarp 7
replum 7
shoot 2390
inflorescence stem 51
node 3
internode 3
stele 3
conducting tissue 3
phloem 3
rosette 2077
stem 3
leaf 1349
petiole 24
juvenile leaf 136
adult leaf 657
senescent leaf 3
cauline leaf 3
shoot apex 129
shoot apical meristem 47
leaf primordia 11
hypocotyl 18
stele 3
conducting tissue 3
xylem 3
cork 3
roots 829
primary root 150
root tip 66
root apical meristem 14
elongation zone 42
maturation zone 27
lateral root 41
created with GENEVESTIGATOR
B.
Dataset: 86 perturbations from data selection: AT_AFFY_ATH1-0
Showing 4 measure(s) of 4 gene(s) on selection: AT-0
CHS AT1G12530 RPP4 SNC1
74 of 672 perturbations fulfilled the filter criteria
Arabidopsis thaliana (74)
<< down-regulated
Fold-Change
up-regulated >>
Filter values for CHS
| 2 |
0.001
Log2-ratio
Fold-Change
p-value
Chemical
norflurazon study 2 (Col-0) / untreated seedlings (Col-0) 1.16 2.24 <0.001
sulfamethoxazole + sucrose (dark) / dark study 13 2.25 4.71 <0.001
Hormone
ABA study 7 (Col-0) / solvent treated guard cell samples (Col-0) 5.72 59.52 <0.001
IAA study 9 (Bay-0) / untreated seedling samples (Bay-0) -3.16 -8.97 <0.001
IAA study 9 (Col-0) / untreated seedling samples (Col-0) -1.02 -2.02 <0.001
zeatin study 2 (Col-0) / solvent treated aerial parts (Col-0) 1.84 3.61 <0.001
Light intensity
dark / 4°C (220-280min) / dark / 21°C (220-280min) 2.20 4.56 <0.001
dark / 4°C (300-360min) / dark / 21°C (300-360min) 2.38 5.22 <0.001
dark / 21°C (60-120min) / moderate light / 21°C (60-120min) -1.43 -2.70 <0.001
high light (Col-0) / low light grown seedlings (Col-0) 1.44 2.71 <0.001
light study 6 (Col-0) / dark grown Col-0 seedlings 2.92 7.60 <0.001
low light / 4°C (140-200min) / low light / 21°C (140-200min) 2.15 4.25 <0.001
low light / 4°C (220-280min) / low light / 21°C (220-280min) 2.65 6.27 <0.001
low light / 4°C (300-360min) / low light / 21°C (300-360min) 3.46 10.91 <0.001
low light study 5 (Col-0) / standard light (Col-0) -2.67 -6.34 <0.001
moderate light / 32°C (60-120min) / moderate light / 21°C (60-120min) -1.14 -2.21 <0.001
moderate light / 32°C (140-200min) / moderate light / 21°C (140-200min) -1.09 -2.13 <0.001
shift dark to light study 2 (polys. RNA) / dark study 6 (polys. RNA) 6.06 67.01 <0.001
shift dark to light study 2 (tot. RNA) / dark study 6 (tot. RNA) 6.49 89.79 <0.001
shift low to high light study 4 (Col-0) / low light study 5 (Col-0) 6.66 99.85 <0.001
shift standard to high light (Col-0) / standard light (Col-0) 3.53 11.37 <0.001
Light quality
blue study 2 / low light grown seedlings (Col-0) 1.21 2.31 <0.001
lincomycin+R+B study 2 (0.5μmol m-2 s-1) / R+B study 2 (0.5μmol m-2 s-1) 2.41 5.21 <0.001
red study 3 (45h) / dark grown seedlings (Col-0) 3.58 11.97 <0.001
red study 4 (18h) / dark study 5 (18h) 2.85 7.34 <0.001
shift lincomycin+R+B 0.5μmol m-2 s-1 to lincomycin+R+B 60μmol m-2 s-1 (1h) ... 1.29 2.47 <0.001
shift lincomycin+R+B 0.5μmol m-2 s-1 to lincomycin+R+B 60μmol m-2 s-1 (4h) ... 3.86 14.19 <0.001
shift lincomycin+R+B 0.5μmol m-2 s-1 to lincomycin+R+B 60μmol m-2 s-1 (24h... 3.54 11.39 <0.001
shift R+B 0.5μmol m-2 s-1 to R+B 60μmol m-2 s-1 (4h) / R+B study 2 (0.5μmol ... 5.42 40.97 <0.001
shift R+B 0.5μmol m-2 s-1 to R+B 60μmol m-2 s-1 (24h) / R+B study 2 (0.5μmol... 5.24 38.14 <0.001
shift UV filtered WG345 to UV filtered WG305 study 2 (Col-0) / UV filtered WG345... 4.76 26.11 <0.001
UV-B (Col-0) / shift LD to CL (Col-0) 3.96 15.54 <0.001
UV filtered WG305 study 3 (Col-0) / UV filtered WG345 (Col-0) 3.58 11.55 <0.001
white + far-red (Col-0) / white study 2 (Col-0) -1.03 -2.04 <0.001
white + far-red study 2 (Col-0) / white study 2 (Col-0) -2.60 -6.06 <0.001
white + far-red study 3 (Col-0) / white study 3 (Col-0) 1.54 2.91 <0.001
Nutrient
glucose (4h) / untreated seedlings 2.08 4.12 <0.001
limiting NH4NO3 / ambient CO2 (midnight) / limiting NH4NO3 / ambient CO2 (... -3.30 -9.85 <0.001
limiting NH4NO3 / elevated CO2 (midnight) / limiting NH4NO3 / elevated CO2 (... -3.04 -8.27 <0.001
P deficiency study 2 (leaf) / Pi supplemented leaf samples 1.80 3.45 <0.001
sucrose / untreated seedlings 1.81 3.53 <0.001
Other
ambient CO2 (mature leaf 10) / ambient CO2 (leaf 10 primordia) -1.82 -3.42 <0.001
germination (12h) / seed desiccation 3.03 8.19 <0.001
germination (12h) / stratification (48h) 3.54 11.70 <0.001
germination (24h) / seed desiccation 5.70 51.90 <0.001
germination (24h) / stratification (48h) 6.21 74.17 <0.001
germination (48h) / seed desiccation 7.31 159.17 <0.001
germination (48h) / stratification (48h) 7.83 227.46 <0.001
Photoperiod
circadian clock study 9 (17h dark+1h light) / circadian clock study 9 (5h dark+1h ... -2.52 -5.73 <0.001
circadian clock study 9 (17h dark+1h light) / circadian clock study 9 (18h dark) 3.65 12.70 <0.001
circadian clock study 9 (18h dark) / circadian clock study 9 (6h dark) -4.91 -30.05 <0.001
night extension (intermediate) / untreated rosette samples -3.30 -9.79 <0.001
night extension (late) / untreated rosette samples -4.83 -28.64 <0.001
Stress
cold study 6 (Coi) / 20°C/18°C treated rosette samples (Coi) 3.08 8.28 <0.001
cold study 6 (Te) / 20°C/18°C treated rosette samples (Te) 2.96 7.55 <0.001
drought study 7 (Col-0) / untreated plant samples (Col-0) -1.54 -2.92 <0.001
drought study 11 (Col-0) / mock treated seedling samples (Col-0) 3.25 9.49 <0.001
heat study 9 (photosynthesis 20% inhibited) / shift 22°C to optimal temperature -1.64 -3.07 <0.001
heat study 9 (photosynthesis 30% inhibited) / shift 22°C to optimal temperature -1.41 -2.65 <0.001
light/drought (Col-0) / untreated leaf samples (Col-0) 5.26 42.08 <0.001
Temperature
10°C / long day (Ler-0) / long day study 2 (Ler-0) 4.01 16.12 <0.001
shift 22°C to optimal temperature / 22°C -1.05 -2.08 <0.001
<16 -12 -8 -4 0 4 8 12 16<
created with GENEVESTIGATOR
C.
Dataset: 14 perturbations from data selection: AT_AFFY_ATH1-0
Showing 4 measure(s) of 4 gene(s) on selection: AT-0
CHS AT1G12530 RPP4 SNC1
12 of 108 perturbations fulfilled the filter criteria
Arabidopsis thaliana (12)
<< down-regulated
Fold-Change
up-regulated >>
Filter values for CHS
| 1.5 |
0.01
Log2-ratio
Fold-Change
p-value
Biotic
G. cichoracearum study 2 (18h) / non-infected whole rosette samples (Col-0) -2.23 -4.61 <0.001
G. cichoracearum study 2 (36h) / non-infected whole rosette samples (Col-0) -4.66 -24.32 <0.001
G. cichoracearum study 2 (96h) / non-infected whole rosette samples (Col-0) -2.08 -4.14 <0.001
G. orontii (72h) / mock treated Col-0 leaf samples (72h) -1.76 -3.48 0.009
G. orontii study 2 (Col-0) / untreated rosette leaf samples (Col-0) 2.39 4.92 <0.001
G. orontii study 6 (Col-0) / untreated rosette leaf samples (Col-0) -2.11 -4.33 0.003
L. huidobrensis (Col-0) / untreated rosette leaf samples (Col-0) -2.45 -5.64 0.004
P. cucumerina (Col-0) / mock inoculated rosette samples (Col-0) -1.25 -2.36 0.005
P. cucumerina study 2 (Col-0) / mock inoculated rosette samples (Col-0) -1.23 -2.33 0.004
P. infestans (24h) / mock treated leaf samples (24h) -1.40 -2.64 <0.001
P. syringae pv. maculicola (Col-0) / mock treated leaf samples (Col-0) -5.21 -37.82 <0.001
Elicitor
HrpZ (4h) / H2O treated leaf samples (4h) -2.18 -4.46 <0.001
<16 -12 -8 -4 0 4 8 12 16<
created with GENEVESTIGATOR
D.
Dataset: 2 perturbations from data selection: AT_AFFY_ATH1-0
Showing 4 measure(s) of 4 gene(s) on selection: AT-0
CHS AT1G12530 RPP4 SNC1
1 of 108 perturbations fulfilled the filter criteria
Arabidopsis thaliana (1)
<< down-regulated
Fold-Change
up-regulated >>
Filter values for AT1G12530
| 1.5 |
0.05
Log2-ratio
Fold-Change
p-value
Elicitor
FLG22 + GA (1h) / untreated leaf disc samples (Ler) -0.81 -1.73 0.012
<16 -12 -8 -4 0 4 8 12 16<
created with GENEVESTIGATOR
