# Supplementary material for: Identification of MOS9 as an interaction partner for chalcone synthase in the nucleus
Source: PeerJ. 2018 Sep 19;6:e5598. doi: 10.7717/peerj.5598 (PMC6151112; doi:10.7717/peerj.5598)
Supplement: Supplemental Information 4 [file peerj-06-5598-s004.docx]

**Table S1.** Oligonucleotide primers used in this study

| **Primer Target** | **Sequence** |
| --- | --- |
| *MOS9* coding forward | CACCATGATGGGTATGGCGAGGAAG |
| *MOS9* coding reverse | GCCAAAGCCAGGAGGGAGTTC |
| *MOS9* coding forward FLIM FRET | GGATCCATGATGGGTATGGCGAGGAAG |
| *MOS9* coding reverse FLIM FRET | GGATCCTGCCAAAGCCAGGAGGGAGTTCAG |
| *MOS9* gene specific SAIL forward | GTCAGGATTAGGTCTGACTTTC |
| *MOS9* gene specific SAIL reverse | CTTTCAACACTACAACAGTACACACG |
| SAIL LB1 | GCTTCCTATTATATCTTCCCAAATTACC |
| **qRT-PCR** | |
| *GAPDH* forward | TTGGTGACAACAGGTCAAGCA |
| *GAPDH* reverse | ACAAACTTGTCGCTCAATGCAATC |
| *UBC* forward | CTGCGACTCAGGGAATCTTCTAA |
| *UBC* reverse | TTGTGCCATTGAATTGAACCCTC |
| *MOS9* forward | GCGGAACATCCGCTAGAGAAATG |
| *MOS9* reverse | GGACTGGTGTTGAAATCAGTTCTTGC |
| *CHS* forward | TGTCTGCCGCTCAGACCATC |
| *CHS* reverse | GAGACCAACTTCCCTCAAATGTCCG |
| *At1g12540* forward | CGTTCCTCGGCAGATCACG |
| *At1g12540* reverse | GCGAGTAGTGACTCTTGAGG |
