# Supplementary material for: Identification of MOS9 as an interaction partner for chalcone synthase in the nucleus
Source: PeerJ. 2018 Sep 19;6:e5598. doi: 10.7717/peerj.5598 (PMC6151112; doi:10.7717/peerj.5598)
Supplement: Supplemental Information 5 [file peerj-06-5598-s005.docx]

| **Table S2**. MarkerLynx XS processing parameters | | |  |  |  |  |
| --- | --- | --- | --- | --- | --- | --- |
|  |  |  |  |  |  |  |
| Retention time range | 0.7-12.0 min |  |  |  |  |  |
| Mass range | 100-1800 m/z |  |  |  |  |  |
| Mass tolerance | 0.02 Da |  |  |  |  |  |
| Xic window | 0.02 Da |  |  |  |  |  |
| Apex Track Peak Parameters | Automatic |  |  |  |  |  |
| Apply Smoothing | Yes |  |  |  |  |  |
| Mass window | 0.02 Da |  |  |  |  |  |
| Retention time window | 0.2 Minutes |  |  |  |  |  |
| Noise elimination level | 10 |  |  |  |  |  |
| Deisotope data | Yes |  |  |  |  |  |
|  |  |  |  |  |  |  |
| Features discovered | 502 |  |  |  |  |  |
|  |  |  |  |  |  |  |
| Statistical analyses (PCA and OPLS-DA) were performed with Pareto scaling of the dataset | | | | | | |
