# Supplementary material for: Identification of MOS9 as an interaction partner for chalcone synthase in the nucleus
Source: PeerJ. 2018 Sep 19;6:e5598. doi: 10.7717/peerj.5598 (PMC6151112; doi:10.7717/peerj.5598)
Supplement: Supplemental Information 7 [file peerj-06-5598-s007.docx]

**Table S4:** Levels of flavonoids detected in *de35S::MOS9::YFP*, *mos9-2,* and *tt4-11* seedlings relative to wild-type Col-0

Putative Identity Retention Detected Ratio of Change (p-value)^a,b^

Time Mass *de35S::* Diagnostic fragments from

(M-H)^-^ *MOS9::YFP mos9-2 tt4-11* high energy function MS^E^

**Kaempferol derivatives:**

-Deoxyhexose 6.54 431.0969 **-1.5 (<0.01) 1.2 (0.01) n.d.** 431.0978, 285.0390

-Hexose-Hexose 5.15 609.1451 **-1.3 (<0.01)** -1.1 (0.27) **n.d.** 609.1460, 285.0410, 283.0248

-Hexose-Deoxyhexose

(Isomer 1) 4.95 593.1506 **1.2 (<0.01)** 1.1 (0.18) **n.d.** 593.1507, 447.0926, 285.0396, 283.0247, 255.0293

(Isomer 2) 5.66 593.1506 1.1 (0.07) **1.3 (<0.01) n.d.** 593.15075, 285.0390

-Deoxyhexose-Deoxyhexose 5.42 577.1557 **1.2 (<0.01) 1.2 (<0.01) n.d.** 577.1551, 431.0966, 285.0395,

283.0245, 255.0298

-Hexose-Hexose-Hexose 3.95 771.1985 **1.2 (<0.01)** 1.1 (0.22) **n.d.** 771.2003, 609.1464, 285.0388,

283.0231

-Hexose-Hexose-Deoxyhexose 4.75 755.2024 **1.6 (<0.01) 1.4 (0.03) n.d.** 755.205, 609.1460, 447.093,

285.0406

-Hexose-Deoxyhexose-Deoxyhexose

(Isomer 1) 4.22 739.2081 **1.1 (0.03) 1.2 (<0.01) n.d.** 739.2089, 593.1516, 431.097,

285.0401, 283.0242, 255.0294

(Isomer 2) 4.91 739.2080 **1.2 (<0.01)** 1.1 (0.06) **n.d.** 739.2094, 593.1512, 447.093,

431.097, 285.040, 283.0248,

255.0297

**Quercetin derivatives:**

-Hexose 5.35 463.0871 **-1.5 (<0.01) -1.2 (<0.01) n.d.** 463.0881, 300.0269, 299.0193,

271.0247

-Hexose-Hexose 3.56 625.1397 **-1.1 (<0.01)** -1.1 (0.16) **n.d.** 625.1384, 301.0322, 299.0150,

271.0242

-Hexose-Deoxyhexose 4.54 609.1454 1.0 (0.92) 1.0 (0.81) **n.d.** 609.1458, 463.0872, 446.0840,

447.0906, 301.0344, 299.0190,

271.0238

-Hexose-Deoxyhexose-Deoxyhexose

(Isomer 1) 3.95 755.2030 1.0 (0.74) **1.2 (<0.01) n.d.** 755.203, 609.145, 447.091,

446.085, 301.034, 300.28,

299.019, 271.0233

(Isomer 2) 4.50 755.2027 1.0 (0.26) 1.0 (0.70) **n.d.** 755.203, 609.145, 463.088,

301.034, 300.028, 299.019,

271.0241

**Isorhamnetin derivatives:**

-Hexose-Deoxyhexose 5.06 623.1608 1.0 (0.87) -1.1 (0.06) **n.d.** 623.1616, 477.0640, 315.0507,

313.0348

^a^ Statistically-significant values are highlighted in bold type.

^b^ n.d. = not detected
